# Co-Occurrence of Intimate Partner Violence Against Mothers and Maltreatment of Their Children With Behavioral Problems in Eastern Europe

**DOI:** 10.1177/10778012231188090

**Published:** 2023-07-20

**Authors:** Antonia Brühl, Catherine L. Ward, Jamie M. Lachman, Heather M. Foran, Marija Raleva, Adriana Baban, Nina Heinrichs

**Affiliations:** 1 Department of Psychology, Clinical Psychology, and Psychotherapy, 9168 University of Bremen, Bremen, Germany; 2 Department of Psychology and Safety and Violence Initiative, University of Cape Town, Rondebosch, Western Cape, South Africa; 3 Centre for Evidence-Based Intervention, Department of Social Policy and Intervention, 6396 University of Oxford, Oxford, UK; 4 MRC/CSO Social and Public Health Sciences Unit, 3526 University of Glasgow, Glasgow, UK; 5 Centre for Social Science Research, University of Cape Town, Rondebosch, Western Cape, South Africa; 6 Department of Health Psychology, 27256 University of Klagenfurt, Klagenfurt, Carinthia, Austria; 7 Department of Psychiatry and Medical Psychology, Medical Faculty, 63579 University of Skopje, Skopje, North Macedonia; 8 Department of Psychology, Babeș-Bolyai University, Cluj County, Cluj-Napoca, Romania

**Keywords:** child maltreatment, intimate partner violence, family violence, low-and middle-income countries, Eastern Europe

## Abstract

This study investigates the co-occurrence of intimate partner violence (IPV) against mothers and their risk of perpetrating child maltreatment (CM) in North Macedonia, the Republic of Moldova, and Romania. Risk factors for IPV, CM, and their co-occurrence were identified. Two samples (*N*_1_*_ _= *112, *N*_2_*_ _* = 701) of mothers with children with behavioral problems were assessed. IPV was reported by 64% of mothers, CM by 96%, and their co-occurrence by 63%. Mothers exposed to emotional IPV reported more physical and emotional CM. Mothers exposed to physical IPV reported more physical CM. Motheŕs own history of CM and offspring's behavior problems were associated with IPV and CM co-occurrence.

Intimate partner violence (IPV) and child maltreatment (CM) are global public health problems (García-Moreno et al., 2013; Hillis et al., 2016). IPV is defined as “behavior by an intimate partner or ex-partner that causes physical, sexual or psychological harm, including physical aggression, sexual coercion, psychological abuse and controlling behaviors” (World Health Organization, 2021, p. 1). For children living in these households, this often means that they see, hear, or are aware of how at least one caregiver harms or threatens the other one (McDonald et al., 2006). Beyond passive witnessing of IPV, children can also experience IPV by becoming involved in the incident or by being aware of it, even if they do not witness the incident (Devaney, 2015). In addition to exposure to IPV, about half of these children may additionally experience CM, including physical abuse (e.g., corporal punishment, such as spanking), emotional abuse, sexual abuse, or neglect (Hamby et al., 2010; Zolotor et al., 2007). Although it is common that both caregivers abuse each other (Slep & O’Leary, 2005; Straus & Gelles, 1990), more severe acts of violence and more severe health outcomes are associated with IPV against women, compared to IPV against men (Caldwell et al., 2012; Chan, 2011; Swan et al., 2008). Therefore, the present study focuses on physical and emotional IPV against mothers and their risk of perpetrating CM in the form of emotional CM, physical CM, and neglect.

## Prevalence Rates for IPV, CM, and Co-Occurrence of IPV and CM

Although children’s exposure to family violence has received increasing research attention over the last 20 years, much of this evidence has been derived from high-income countries. Evidence from low- and middle-income countries (LMICs) is rare (Kyegombe et al., 2015), especially in Eastern European countries “with high levels of inequality and where there are few social safeguards to buffer families from economic stress” (Sethi et al., 2013, p. 9). Several studies show that families characterized by social and economic disadvantage are at a higher risk for both IPV and CM (e.g., Finkelhor et al., 2007; Guedes et al., 2016). In Eastern Europe, physical CM and IPV towards the mother are two of the most frequently reported adverse childhood experiences (ACEs; Bellis et al., 2014). ACE studies in Romania (*N* = 2,088), North Macedonia (*N* = 664), and the Republic of Moldova (*N* = 1,534) found childhood lifetime prevalence rates ranging from 12% to 27% for physical CM, 11% to 24% for emotional CM, 4% to 13% for sexual CM, 7% to 20% for physical neglect, and 13% to 31% for emotional neglect. Regarding the co-occurrence of ACEs in these countries, 10% to 17% of youth reported witnessing violence against their mother with co-occurrence rates of 32% to 39% for physical CM and 20% to 44% for emotional CM of those who also witnessed IPV (Baban et al., 2013; Lesco et al., 2018; Raleva et al., 2013). Rates found in ACE studies are provided by country in the supplementary material.

There are several nationally representative and often cited studies from the United States about the co-occurrence of IPV and CM (Hamby et al., 2010; McGuigan & Pratt, 2001; Zolotor et al., 2007), but we could identify only one such study in Eastern Europe with 208 Kosovar adolescents (Kelmendi et al., 2019). Finding similar results to other studies, mothers exposed to physical IPV were four times more likely to report severe physical CM (OR_adj _= 3.52). To our knowledge, no previous study has investigated the likelihood of experiencing CM among families exposed to IPV in North Macedonia, Republic of Moldova, or Romania.

## Co-Occurrence and Shared Risk Factors

IPV and CM are two forms of ACEs which often occur together and share common risk factors. They also share common short- and long-term consequences for children, including behavioral problems, trauma-related symptoms, depression, anxiety, alcohol or drug use, and re-victimization in later life (Evans et al., 2008; Gilbert et al., 2009). In many IPV cases, the perpetrator does not assault only their partner, but rather several family members (Hamby et al., 2010), which may be partially explained by perpetrators’ disposition and person-based risk-factors (Knickerbocker et al., 2007). Person-based risk-factors for IPV and CM include impulsivity, genetic loading for aggression, stress reactivity, young age, mental health problems, substance use, and CM experiences during the perpetrators’ own childhood (Guedes et al., 2016; Jouriles et al., 2008; Slep & O’Leary, 2001). Increasing attention to perpetrators’ own history of CM and IPV has generated evidence supporting an intergenerational transmission of violence, often referred to as the cycle of violence. According to the initial theory (Kalmuss, 1984) and findings from several meta-analyses, exposure to CM and/or IPV in childhood may increase the likelihood of IPV victimization or perpetration (Godbout et al., 2019; Li et al., 2019) and perpetrating CM against their children later in life (Assink et al., 2018), which may predispose children to victimization or perpetration of violence in the future.

In addition to person-based risk factors, several environmental and contextual risk factors have been suggested to cause stress in the family system, which increases the potential for aggressive behavior across family members (Knickerbocker et al., 2007). Guedes et al. (2016) reviewed 48 sources (including reviews and meta-analyses) in LMICs on the intersections between violence against women and violence against children. External environmental stressors found to be related to IPV and CM include poverty, gender inequality and discrimination, living in a violent community, lack of responsive institutions, and weak legal sanctions against violence. Contextual stressors within the family include financial stress, parenting stress, child behavior problems, male dominance in the household, unemployment, the presence of a non-biological father figure at home, and marital conflict (Guedes et al., 2016; Jouriles & Norwood, 1995; Jouriles et al., 2008; Margolin & Gordis, 2003). Only a few studies have identified unique risk factors of co-occurrence by investigating which risk factors distinguish amongst caregivers who perpetrate either IPV or CM (Nichols & Slep, 2022). Unique risk factors of co-occurrence found in previous work include younger children, caregiver history of CM during childhood, depression, adult or adolescent substance abuse, violent and non-violent convictions, current relationship difficulties, dramatic and emotional Cluster B personality traits, and parenting stress (Dixon et al., 2007; Nichols & Slep, 2022; Slep & O’Leary, 2009; Tajima, 2004).

Several studies have investigated rates and risk factors for either IPV or CM, but they tend to do so without considering the relationships between the two (Bidarra et al., 2016). Moreover, key limitations in previous studies include that most evidence is derived from high-income settings, “where social and gender norms, overall burden of co-occurring IPV and violence against children, and structural adversities differ from other contexts” (Namy et al., 2017, p. 41). IPV and CM are not only more prevalent in LMICs (García-Moreno et al., 2013; Hillis et al., 2016), but growing up in resource-poor contexts is linked to several risk factors for childreńs mental health that may further increase parenting difficulties (Kieling et al., 2011; Ward et al., 2016). There is compelling reason to posit that children's mental health problems, such as externalizing behavior, are not only well-known consequences of CM and parental IPV exposure (Gilbert et al., 2009; Vu et al., 2016), but also constitute a risk factor for both forms of family violence (Choe et al., 2013; Combs-Ronto et al., 2009; Pu & Rodriguez, 2021; Stith et al., 2009). Thus, children growing up in LMICs who show behavioral problems are a particularly vulnerable population for exposure to multiple forms of family violence. Among the few English-language studies examining IPV and CM in Eastern Europe, none of these investigated the rates and odds of co-occurrence in a sample of mothers with children showing behavioral problems. Moreover, no other study has explored overlapping specific risk factors in North Macedonia, the Republic of Moldova, or Romania, which is crucial in developing prevention and intervention approaches and strengthening the local public health agenda in these countries.

To contribute to a more integrated understanding of family violence across the range of income, the first aim of this study is to investigate intersections across types of IPV victimization and CM perpetration in three understudied LMICs: North Macedonia, the Republic of Moldova, and Romania. We aimed to (a) describe the rates of IPV victimization, CM perpetration, and their co-occurrences in two independent samples of mothers of young children with elevated behavioral problems, and (b) explore the risk for CM perpetration of mothers exposed to IPV victimization during the last month. The second aim was to identify risk factors for IPV, CM, and risk factors that are *specifically* associated with the co-occurrence of IPV and CM compared with mothers reporting either IPV or CM. We anticipate that the common person-based risk factors stated in the literature (i.e., mother’s young age, mental health problems, alcohol use, and own history of CM), contextual risk factors (i.e., unemployment, child behavior problems, and parenting stress), and environmental risk factors (i.e., household poverty) will be associated with a higher co-occurrence of IPV and CM.

## Method

### Study Design

The present study is embedded in a larger prevention trial that aims to adapt, optimize, and test the Parenting for Lifelong Health program for parents of young children ages 2 to 9 years (PLH-YC) in North Macedonia, the Republic of Moldova, and Romania (Frantz et al., 2019; Lachman et al., 2019). We applied the Multiphase Optimization Strategy (MOST; Collins, 2018), which is a methodological approach for developing behavioral interventions. The MOST comprises three phases, which were sequentially implemented as follows: The Preparation Phase (Phase 1) involved a single-arm pre–post pilot study design to investigate the implementation feasibility and acceptability of the adapted intervention. Building on the results from the preceding pilot study, the Optimization Phase (Phase 2) aimed to optimize the intervention package by identifying the most cost-effective intervention components using a clustered factorial experimental design (Lachman et al., 2019). In the Evaluation Phase (Phase 3), the optimized intervention will be tested in a randomized controlled trial. Only Phase 1 and Phase 2 baseline data were used in this cross-sectional investigation. Information on the intervention and Phase 3 is published elsewhere (Taut et al., 2021). Phase 1 was conducted between April and June 2018. Trained data assessors obtained informed consents, screened caregivers for eligibility, and completed pre-assessments by using computer-assisted self-interviewing (“CASI”) method. Assessments were delivered at a venue suitable for the family (e.g., at home, health centers, kindergartens, or schools). Pre-assessments comprised self-report questionnaires on demographics, child and parental mental health, and parenting behavior. After participating in the 12-session version of PLH-YC, post-assessments were conducted approximately 16 to 20 weeks after baseline assessment (data not used in this study). Caregivers received a food voucher or hygiene products (worth approximately 5€) as compensation for each assessment.

In Phase 2, caregivers completed pre-assessments between March and April 2019, and subsequently participated in experimentally varied versions of PLH-YC over 10 weeks. Data assessment procedures were similar to Phase 1 with post-assessments conducted seven months after baseline and follow-up assessments conducted 11 months after baseline. Caregivers also received a food or gift voucher for each assessment point (worth approximately 2–5 €). The study protocols for Phases 1 and 2 were approved by the ethics committee of the Klagenfurt University (approval number: 2018-021) as well as the local committees in the three country sites (approval numbers: 03-1460/11; nr. 43 la nr. 56; 3533/05.03.2018).

### Participants

The present study included data collected from two separate samples of biological mothers, each recruited for the first two (of three) project phases reflected in MOST (Collins, 2018). Data from *N*_1_ = 112 mothers who participated in the pilot study (Phase 1; Frantz et al., 2019) and data from *N*_2_ = 701 mothers collected in the optimization study (Phase 2; Lachman et al., 2019) were used for this cross-sectional investigation. According to the inclusion criteria, all mothers were at least 18 years old, were responsible for a child aged 2 to 9 years old, lived in the same household as the child for at least four nights a week, reported elevated levels of child behavioral problems, and provided written consent. Subclinical levels of child behavior problems were screened with modified versions from the Eyberg Child Behavior Inventory (ECBI; Eyberg & Pincus, 1999; used in Phase 1) and the Child and Adolescent Behavior Inventory oppositional defiant disorder subscale (Burns et al., 2015; used in Phase 2). We excluded caregivers exhibiting severe mental health problems, acute mental disabilities, or who were referred to child protection services due to child abuse.

Participants were recruited through social media and referrals (Phase 1), as well as through health centers, kindergartens, and schools (Phase 2). All participants agreed to participate in the PLH-YC program and provided written consent for participation in the prevention trial. We only used data from biological mothers who reported to be in a relationship to examine IPV victimization and CM perpetration. The mothers lived in North Macedonia (*n*_1_ = 40, *n*_2_ = 239), the Republic of Moldova (*n*_1_ = 33, *n*_2_ = 238), and Romania (*n*_2_ = 39, *n*_2_ = 224). Across countries, the majority of mothers was married and living together with their spouses (76% in Phase 1, 92% in Phase 2) or unmarried and living together (15% in Phase 1, 4% in Phase 2). Table 1 summarizes demographic characteristics.

**Table 1:** Sample Characteristics for the Phase 1 (*N*_1_ = 112) and Phase 2 Study Samples (*N*_2_ = 701).

Variables	Phase 1 (*N *= 112)	Phase 2 (*N* = 701)
	*M* (SD)	*M* (SD)
Age		
Mothers	34.24 (6.13)	35.50 (5.35)
Child	5.75 (2.06)	5.64 (1.96)
Number of children living in the household	2.35 (1.58)	1.81 (0.71)
	*n (%)*	*n (%)*
Gender (female)		
Child	63 (56.3)	280 (39.9)
Education level (no university or college)	56 (50.0)	167 (23.8)
Marital status		
Married and living together	85 (75.9)	644 (91.9)
Married and not living together	8 (7.1)	14 (2.0)
Unmarried living together	17 (15.2)	31 (4.4)
Unmarried not living together	2 (1.8)	12 (1.7)

*Note. M* = Mean; SD = standard deviation.

### Measures

All measures were translated and checked by back-translation if they were not available in the local languages (i.e., Macedonian, Romanian, and Moldovan Romanian). Translations were completed by local clinicians and researchers in the field of psychology who were fluent in English. After back-translation, native English researchers and a native Romanian researcher compared the original items with the back-translated items and minor wording adaptions were made to ensure that the intended meaning remained during the translation process. Afterwards, measures were tested in a feasibility study in each country. The scale properties were examined, including validity correlations and response distributions, with a pilot sample (*N* = 140; Jansen et al., 2021; Williams et al., 2022). Based on the translation processes and the Phase 1 results, all measures for Phase 2 were selected. To address the research questions of the current study, we chose the respective measures from a pool of all measures based on the aforementioned literature review.

#### Demographics

Mothers provided information on their age, child’s age and gender, number of children living in the household, mother’s education, and marital status.

#### Child Maltreatment

To assess mother’s perpetration of CM, an adapted version of the widely used International Society for the Prevention of Child Abuse and Neglect (ISPCAN) Child Abuse Screening Tool-Trial scale was used (ICAST-TC; Meinck et al., 2018; Runyan et al., 2009). Sixteen items were employed to assess the frequency of CM perpetration in the past month (e.g., “In the past 4 weeks, how often did you discipline your child by pinching or shaking him/her?”). Mothers responded on a 9-point scale from 0 to more than 8 times, and how often they maltreated or neglected their offspring. The ICAST-TC has been shown to be sensitive detecting changes in abusive behavior and has demonstrated adequate criterion validity with parents of adolescents (Meinck et al., 2018). An adapted version of the ICAST-TC was translated and tested for this study in three languages. In the initial feasibility study, we evaluated the validity of the measure before use in the larger trial (Frantz et al., 2019; Jansen et al., 2021). The validity was supported by correlations with the Parenting Scale (PS; Arnold et al., 1993), which assesses dysfunctional discipline practices. The overall CM score of the adapted ICAST-TC was significantly associated with an increased total score of the PS (*β* = .24) and an increased subscale score on parental overreactivity (*β* = .38). The subscale physical CM of our measure was significantly correlated with reduced laxness in the PS (*β* = -.18). Dichotomous variables were created (0 = no incidence, 1 = at least one incidence) to assess the incidence of any form of CM, as well as of physical CM, emotional CM, and neglect during the previous month.

#### Intimate Partner Violence

To assess physical and emotional IPV victimization of mothers, we used an adaptation of the Brief Screening Instrument for Partner Maltreatment (Heyman et al., 2013) and the revised Conflict Tactics Scale (CTS2S; Straus et al., 1996). The CTS2S is the short form of the most widely used instrument for assessing IPV and has demonstrated good construct and concurrent validity, similar to the full version (Straus & Douglas, 2004). This tool comprises 29 items assessing emotional and physical aggression as victims and perpetrators during the last month. Mothers rated on a 9-point scale from 0 to 8 (0 = *never happened;* 8 = *more than 8 times*), with an additional response for incidents that occurred during the current relationship but prior to the past month. Given that the IPV victimization and IPV perpetration subscales were highly correlated in both samples (*n*_1_ = 112, rho* *= 0.72, *p* < .001; *n*_2 _= 699, rho = 0.86, *p*  < .001), only the victimization subscales were used, in line with the scope of this study. Dichotomous variables were calculated (0 = *no incidence*, 1 = *at least one incidence*) to estimate the rates of any IPV victimization, physical IPV victimization, and emotional IPV victimization during the last month. Any IPV victimization reported prior to the previous month was used as a control variable. Throughout this study, the co-occurrence of IPV victimization (referred to as IPV) and CM perpetration (referred to as CM) relates to incidents that occurred during the last month to ensure the assessment of incidents within the same reporting period.

#### Person-Based, Environmental, and Conceptual Risk Factors

The Depression Anxiety Stress Scales (DASS; Lovibond & Lovibond, 1995) was used to assess mothers’ mental health (Phase 2 data only was relevant to the current study). On 21 items, mothers reported emotional states of depression, anxiety, and stress during the previous week. Symptom frequency was rated on a 4-point Likert scale (0 = *never*, 1 = *sometimes*, 2 = *often*, 3 = *always*). We found excellent internal reliability at baseline (Cronbach's *α* = .92). Mothers’ history of CM in their families of origin was measured using an adapted version of the ISPCAN Child Abuse Screening Tool Retrospective version (ICAST-R; Dunne et al., 2009). Three items described physical CM, emotional CM, and corporal punishment during the first 18 years of life, to which the mothers responded with *yes* (1) or *no* (0). An acceptable internal consistency was obtained (*α* = .72). The Food Insecurity Experience Scale (FIES 8-item scale; Ballard et al., 2013) was used to assess caregivers’ access to food as a proxy to estimate poverty. For eight items, mothers responded with *yes* (1), *no* (0), or *don’t know* (999). The *don’t know* responses were treated as missing data, and the total sum score was used in our analysis (*α* = .87). The use of alcohol was measured with The Alcohol Use Disorders Identification Test (AUDIT; de Meneses-Gaya et al., 2009), which consists of 10 items assessing frequencies of alcohol-related behaviors. However, the seven problem scale items showed little variability in Phase 1, so only the 3-item consumption subscale (AUDIT-C) was employed in Phase 2. Mothers responded on a 3- or 5-point Likert Scale and the three items were summed up to build a total sum score, which was used in our analysis (*α* = .40). The widely used parent report of the Child Behavior Checklist (Achenbach & Rescorla, 2001) was employed to assess child behavior problems, with 103 items for children aged 1½ to 5 and 113 items for children aged 6 to 18. Mothers responded on a 3-point Likert Scale (0 = *not true* to 2 = *very true*). The *T*-score for the externalizing behavior subscale was used in our analysis. The Parenting Stress Scale (Berry & Jones, 1995) comprises 18 items, which are rated on a 5-point Likert scale (1 = *strongly disagree* to 5 = *strongly agree*) to assess parental stress (*α* = .80). We used the total sum score in our analysis, with higher scores indicating greater levels of parental stress.

### Data Analysis

We tested the data for normality, multicollinearity, linearity, and examined missing data. Missing data in the total samples (Phase 1, *N*_1_* *= 112; Phase 2, *N*_2_ = 701) were mainly due to missing IPV data (*n*_2_ = 2). Thus, almost no unplanned missingness would impact the analyses (<5%). To evaluate country differences in rates, we conducted chi-square analyses with subsequent post hoc tests. Associations between the dependent variables (i.e., any CM, physical CM, emotional CM, neglect) and independent variables (i.e., any IPV, physical IPV, emotional IPV) were tested using logistic regressions in both samples (*n*_1_ = 112, *n*_2_ = 699). Results are reported as adjusted odds ratios (OR_adj_) with 95% confidence intervals (CIs) after controlling for children's age, gender, and country site.

To examine the second aim, only Phase 2 baseline data (owing to the reduced sample size in Phase 1) was used to identify risk factors that are likely to be associated with any IPV, any CM, and the co-occurrence of IPV and CM. Dichotomous dependent variables were created for any IPV during the last month (Model 1, 0 = no IPV, 1 = at least one incidence of IPV), any CM during the last month (Model 2; 0 = no CM, 1 = at least one incidence of CM), and the co-occurrence of any IPV and any CM during the last month (Model 3, 0 = either current CM or IPV, 1 = at least one incidence of both, CM and IPV). We conducted three binary logistic regressions using the forced entry method, with the following independent variables included in each model: mother’s age, mental health problems, alcohol use, history of CM, parenting stress, unemployment, child externalizing behavior problems, and food insecurity. We further controlled for county site, children's age, gender, and IPV reported *prior* to the last month. In model 2, IPV prior to the last month was not entered because of empty cells. Analyses were conducted with reduced sample sizes owing to missing data in the risk factors’ measures. Mothers who reported neither IPV nor CM (*n* = 47) were further excluded from model 3; thus, sample sizes of *n*_2_ = 483 (Model 1), *n*_2 _= 484 (Model 2), and *n*_2_ = 456 (Model 3) remained. Regressions were performed using SPSS v.26 with 1,000 bootstrapped resamples.

## Results

### Rates for IPV and CM

The frequencies and rates are presented in Table 2. Significant differences across countries emerged for all forms of CM, physical IPV, and caregiver’s history of CM in Phase 2. Post hoc comparisons of rates revealed that significantly higher rates of any CM (96%) and emotional CM (96%) were reported by mothers in Romania. Mothers in Moldova reported significantly more physical CM (66%), neglect (19%), and physical IPV (30%). In contrast, significantly lower rates of any CM (85%), physical CM (44%), emotional CM (84%), and physical IPV (14%) were seen among mothers in North Macedonia.

**Table 2:** Frequencies and Rates for CM, IPV, and Mother’s History of CM Separated by Sample and Country.

Variables	Phase 1 (*n_1_* = 112)					Phase 2 (*n_2_* = 701)				
	Total *n(%)*	Macedonia *n(%)*	Moldova *n(%)*	Romania *n(%)*	*p*	Total *n(%)*	Macedonia *n(%)*	Moldova *n(%)*	Romania *n(%)*	*p*
Any CM (past month)^a^	108 (96.4)	40 (100.0)	32 (97.0)	36 (92.3)	.180	641 (91.4)	202 (84.5)	224 (94.1)	215 (96.0)	<.001
Physical CM	81 (72.3)	29 (72.5)	26 (78.8)	26 (66.7)	.519	393 (56.1)	104 (43.5)	157 (66.0)	132 (58.9)	<.001
Emotional CM	107 (95.5)	40 (100.0)	32 (97.0)	35 (89.7)	.078	636 (90.7)	200 (83.7)	221 (92.9)	215 (96.0)	<.001
Neglect	23 (20.5)	7 (17.5)	4 (12.1)	12 (30.8)	.125	97 (13.8)	33 (13.8)	46 (19.3)	18 (8.0)	.002
Any IPV (relationship)^b^	75 (67.0)	29 (72.5)	21 (63.6)	25 (64.1)	.649	451 (64.5)	147 (61.8)	159 (67.1)	145 (64.7)	.478
Any IPV (past month)^a^	72 (64.3)	28 (70.0)	19 (57.6)	25 (64.1)	.544	431 (61.7)	136 (57.1)	154 (65.0)	141 (62.9)	.191
Physical IPV^a^	19 (17.0)	7 (17.5)	5 (15.2)	7 (17.9)	.946	141 (20.2)	34 (14.3)	72 (30.4)	35 (15.6)	<.001
Emotional IPV^a^	71 (63.4)	28 (70.0)	18 (54.5)	25 (64.1)	.392	418 (59.9)	131 (55.0)	147 (62.0)	140 (62.8)	.169
Mother’s history CM	72 (64.9)	22 (55.0)	22 (66.7)	28 (73.7)	.217	458 (66.0)	105 (44.3)	173 (73.0)	180 (81.8)	<.001
Co-occurrence of any IPV^a^ and any CM^a^	71 (63.4)	28 (70.0)	19 (57.6)	24 (61.5)	.524	418 (59.8)	128 (53.8)	151 (63.7)	139 (62.1)	.062

*Note.* CM = child maltreatment; IPV = intimate partner violence victimization.

Missing data on mother’s history of CM (*n_1_*_ _= 1, *n_2_*_ _= 7) led to reduced sample sizes of *n_1_* = 111 and *n_2_* = 694. Country differences were tested using chi-square analysis.

^a^Incidences during the past month.

^b^All previous incidences in the current relationship, including prior to last month*; p* = significance; Macedonia = North Macedonia; Moldova = Republic of Moldova; in Phase 2, missing data on IPV (*n*_2 _= 2) led to a reduced sample size of *n*_2_ = 699 for all IPV scales and co-occurrence of any IPV and any CM.

### Rates and Odds Ratios for Co-Occurrence of IPV and CM

Table 3 contains rates and OR_adj_ for each form of CM comparing mothers exposed to IPV with those not exposed during the last month. In Phase 1, 99% of mothers exposed to any IPV also reported any CM. In Phase 2, 97% of mothers exposed to any IPV in the last month reported at least one incident of any CM in the same month. Regarding the type of IPV in Phase 2, exposure to physical IPV was associated with current physical CM, whereas emotional IPV was associated with physical CM and emotional CM. Any IPV was associated with physical CM and emotional CM.

**Table 3:** Rates and Adjusted Odds Ratios for the Co-Occurrence of IPV Victimization with CM Perpetration: Past Month Reports Separated by Sample.

	Any IPV			Physical IPV			Emotional IPV		
Phase 1	%	OR_adj_ [95% CI]	*p*	%	OR_adj_ [95% CI]	*p*	%	OR_adj_ [95% CI]	*p*
Any CM	98.6	5.86 [0.55, 62.34]	.143	100	–	–	98.5	5.66 [0.53, 60.46]	.152
Physical CM	73.6	1.27 [0.53, 3.06]	.589	63.2	0.64 [0.22, 1.86]	.411	74.6	1.48 [0.62, 3.55]	.379
Emotional CM	98.6	9.68 [0.92, 102.48]	.059	100	–	–	98.6	9.41 [0.88, 100.36]	.063
Neglect	15.3	0.38 [0.15, 1.01]	.052	26.3	1.50 [0.46, 4.86]	.498	15.5	0.39 [0.15, 1.04]	.060
Phase 2	%	OR_adj_ [95% CI]	*p*	%	OR_adj_ [95% CI]	*p*	%	OR_adj_ [95% CI]	*p*
Any CM	97.0	7.02 [3.66, 13.46]	**<**.**001**	96.5	2.57 [0.99, 6.69]	.052	96.9	6.50 [3.39, 12.47]	**<**.**001**
Physical CM	68.7	3.99 [2.87, 5.57]	**<**.**001**	80.1	3.59 [2.27, 5.66]	**<**.**001**	68.2	3.60 [2.59, 4.99]	**<**.**001**
Emotional CM	96.8	7.04 [3.75, 13.22]	**<**.**001**	96.5	2.88 [1.11, 7.44]	.030	96.7	6.49 [3.46, 12.20]	**<**.**001**
Neglect	16.2	1.81 [1.11, 2.93]	.017	20.6	1.65 [1.00, 2.72]	.049	16.0	1.76 [1.09, 2.83]	.020

*Note.* CM = child maltreatment perpetration during last month; IPV = intimate partner violence victimization during last month; % = percentage of IPV-exposed mothers reporting CM during the last month; OR_adj_ = adjusted odds ratio after controlling for country site, child's age and gender; *CI *= 95% CI for odds ratio; *p*= significance (in bold significant with adjusted alpha = .004); Emdash indicates that OR_adj_ is not available due to empty cell; Phase 2 (*n_2_* = 699) has a reduced sample size due to missing data (*n*_2_ = 2).

### Risk Factors for IPV, CM, and the Co-Occurrence of IPV and CM

The results of the binary logistic regressions can be found in Table 4, which provides the coefficients, Wald statistics (W*χ*^2^), and OR_adj _ for each predictor. Model 1 significantly predicted any IPV in the past month (omnibus *χ*^2 ^= 71.86, df = 13, *P* < .001; Nagelkerke's *R*^2 ^= .19), with any IPV associated with caregiver’s mental health problems, a caregiver’s history of CM, and externalizing behavioral problems of the child being associated. In Model 2 (omnibus *χ*^2 ^= 81.94, df = 12, *P* < .001; Nagelkerke's *R*^2^*^ ^* = .38), externalizing behavior problems of the child were significantly associated with any CM during the last month. Model 3 (omnibus *χ*^2 ^= 67.54, df = 13, *P* < .001; Nagelkerke's *R*^2 ^= .19) revealed that a mother’s history of CM and externalizing behavioral problems of the child were significantly associated with the co-occurrence of any IPV and any CM during the last month.

**Table 4:** Risk Factors for IPV, CM, and the Co-Occurrence of IPV and CM in the Phase 2 Study Sample.

	Any IPV				Any CM				Co-occurrence of IPV and CM			
Variables	*B*	Wχ^2^	*p*	OR_adj_ [95% CI]	*B*	Wχ^2^	*p*	OR_adj_ [95% CI]	*B*	Wχ^2^	*p*	OR_adj_ [95% CI]
Age	−0.01	0.02	.893	1.00 [0.96, 1.04]	−0.08	2.86	.062	0.93 [0.85, 1.01]	−0.02	0.43	.563	0.99 [0.94, 1.03]
Mental health	0.02	7.65	.**017**	1.02 [1.01, 1.04]	−0.01	0.07	.806	1.00 [0.96, 1.03]	0.02	5.40	.041	1.02 [1.00, 1.04]
Alcohol use	0.20	2.67	.106	1.22 [0.96, 1.55]	0.56	4.01	.028	1.75 [1.01, 3.03]	0.19	2.24	.118	1.21 [0.94, 1.54]
History of CM	0.66	7.49	.**004**	1.93 [1.20, 3.08]	1.11	5.63	.022	3.04 [1.21, 7.64]	0.61	5.95	.**015**	1.85 [1.13, 3.02]
Parenting Stress	0.01	0.24	.630	1.01 [0.98, 1.04]	0.01	0.12	.698	1.01 [0.95, 1.08]	0.02	0.90	.378	1.02 [0.98, 1.05]
Unemployment	−0.59	3.70	.056	0.55 [0.30, 1.01]	−0.12	0.03	.858	0.89 [0.24, 3.36]	−0.72	5.03	.027	0.49 [0.26, 0.91]
Externalizing behavior	0.03	9.45	.**003**	1.03 [1.01, 1.06]	0.11	13.54	.**005**	1.12 [1.06, 1.19]	0.04	10.18	.**007**	1.04 [1.01, 1.06]
Food insecurity	−0.03	0.06	.761	0.97 [0.80, 1.19]	−0.03	0.01	.868	0.97 [0.60, 1.58]	−0.02	0.02	.883	0.99 [0.80, 1.22]

*Note.* IPV = Intimate partner violence victimization during last month; CM = Child maltreatment perpetration during last month; *B* = unstandardized regression coefficient; Wχ^2 ^= Wald statistic; *p* = significance (in bold significant with adjusted alpha = .017); OR_adj_ = Adjusted odds ratio; CI* *= 95% CI for odds ratio; Caregiver’s mental health problems assessed with the DASS (Lovibond & Lovibond, 1995); Caregiver’s Alcohol use assessed with AUDIT (de Meneses-Gaya et al., 2009); Caregiver’s history of CM assessed with ICAST-R (Dunne et al., 2009; 0 = no history of CM, 1 = history of CM); Parenting Stress assessed with Parenting Stress Scale (Berry & Jones, 1995); Unemployment (0 = currently working, 1 = currently not working); Child externalizing behavior assessed with externalizing subscale of the CBCL (Achenbach & Rescorla, 2001); Food insecurity assessed with the FIES (Ballard et al., 2013).

## Discussion

Two key findings related to IPV and CM rates emerged. First, in our samples of mothers with children showing elevated behavioral problems, family violence was not only widespread but more likely to be the rule rather than the exception across countries. More than half of the mothers reported exposure to IPV during the past month, almost every mother reported perpetration of at least one incident of CM within the same month, and approximately two-thirds reported their own history of experiencing CM as a child. Second, our findings are in line with previous studies showing that caregivers exposed to IPV are more likely to report CM (Hamby et al., 2010; Zolotor et al., 2007).

### Intimate Partner Violence and Co-Occurrence of CM

The frequency of IPV identified in the present study is very similar to that of ACE and other studies, indicating roughly about 60% emotional IPV and 20% physical IPV (Baban et al., 2013; Kelmendi et al., 2019; Lesco et al., 2018; Raleva et al., 2013). Mothers exposed to any IPV were seven times more likely to show any form of CM within the past month than mothers who were not exposed, which is in line with previous findings on past year reports in other more western regions (e.g., OR_adj_ = 5.32; Hamby et al., 2010). Mothers who reported physical IPV in the past month were 3.9 times more likely to report physical CM in the past month than those who did not report physical IPV, which is similar to previous studies using past-year parental reports (OR_adj_ = 2.57; Zolotor et al., 2007) and past-year child reports (OR_adj_ = 5.03; Hamby et al., 2010, USA; OR_adj_ = 3.52; Kelmendi et al., 2019, Kosovo). Our co-occurrence rates of physical IPV and current physical and emotional CM are considerably higher than the estimates in the ACE studies (32%–39% physical CM, 20%–44% emotional CM, and 21%–33% neglect), which is more in line with the recent study from Kosovo (Kelmendi et al., 2019). However, our rates of co-occurrence are less surprising, when considering the high rates of CM that we found and the sampling of families with children with elevated behavioral problems.

### Physical Child Maltreatment

In line with previous studies from LMICs (e.g., Kumar et al., 2019; Petrovic et al., 2016), more than half of the mothers (56%–72%) reported perpetrating at least one incident of physical CM in the form of slapping, spanking, pushing, pinching, shaking, or disciplining the child with an object (e.g., stick or belt) during the last month. The ACE studies (Baban et al., 2013; Lesco et al., 2018; Raleva et al., 2013) found lower prevalence rates of physical CM in Romania (27%), North Macedonia (21%), and the Republic of Moldova (12%). A potential explanation for this is that we applied a broader definition of physical CM. In the ACE studies, physical CM and corporal punishment (including spanking) were assessed separately. More compatible with our estimates, they found that 20% to 72% respondents reported corporal punishment across the three countries. However, the high rates that we found are particularly remarkable given that we used caregiver self-reports (as opposed to the ACE studies). This may reflect an overall high level of endorsement of physical punishment in these countries. The variety of definitions and measures used to assess CM makes it difficult to compare prevalence rates across studies, emphasizing the need for a more common consensus on constructs in this research field. Moreover, our sampling of families with children with elevated behavior problems may have resulted in higher rates of CM, especially given the strong associations between the two factors.

In our study, the proportion of physical CM was significantly lower in North Macedonia than in the other countries. Sebre et al. (2004) compared child reports of CM across Eastern European countries and also found lower rates of CM in North Macedonia (compared to Latvia, Lithuania, or Moldova). The Macedonian researchers assumed that caregivers were not less abusive, but that children growing up in North Macedonia may have more internalized “sociocultural prohibitions against speaking badly of one's family” (Sebre et al., 2004, p. 123). Therefore, the children in their study may have withheld information on maltreatment by their parents. Mothers in our study may have had more hesitations to provide information that could cast a bad light on the family system and indicate difficulties in their mother–child relationship.

### Emotional Child Maltreatment

Almost every mother in our samples reported perpetrating at least one incident of emotional CM within the last month (91%–96%). The ACE studies found considerably lower rates for emotional CM, ranging from 11% to 24% across the three countries (Baban et al., 2013; Lesco et al., 2018; Raleva et al., 2013). Our 1-month prevalence rates for emotional CM further exceeded the lifetime estimates found in studies that used the child version of the ICAST measure in samples from India (73%; Kumar et al., 2019), across nine Balkan countries (64%–83%; Nikolaidis et al., 2018, BECAN study), and Saudi Arabia (75%; Al-Eissa et al., 2016). One may argue that these discrepancies may, in part, be explained by relying on past month parent reports instead of retrospective child reports. In the ACE studies, young adults retrospectively reported on their first 18 years of life. In the BECAN study, children aged 11 to 16 years provided information on lifetime and last year prevalence rates (Nikolaidis et al., 2018). They found throughout higher rates than the ACE studies and, thus, were more comparable to our rates. These discrepancies across studies indicate the strong need to include both perspectives, the parent and the child report, in future studies.

Furthermore, we applied a low threshold for CM across physical CM, emotional CM, and neglect (i.e., at least one incidence in the past month). Thus, one item (“How often did you shout, yell or scream at your child”?) extensively contributed to the high rates found in our study, but, in contrast, was not considered in the figures for emotional abuse in the ACE studies. However, a meta-analysis of prevalence rates for emotional abuse reported estimates (parent and child reports) ranging from 35% to 79% around the globe (Stoltenborgh et al., 2012), but found no differences in rates between studies using broader or narrower definitions of emotional CM. Even studies from LMICs that assessed yelling at the child within the construct of emotional abuse (e.g., Kelmendi et al., 2019; Kumar et al., 2019) did not find prevalence rates nearly as high as we did, so that our findings indicate that yelling was extraordinarily common in our sample. Based on our findings, we assume that yelling can be considered a parenting approach to child discipline commonly used in North Macedonia, the Republic of Moldova, and Romania. As yelling and other verbal punishments are acceptable and even expected in some societies, emotional CM appears to be the type of CM that varies most across cultures (Slep et al., 2011). Researchers have argued that although yelling may be considered as common parenting behavior, it can also be defined as a verbally abusive parenting practice (Polcari et al., 2014). Finally, we recruited families to participate in a parenting intervention so that our selected samples were characterized by elevated child behavior problems. The high rates may be partly explained by the interplay between child behavior problems and dysfunctional parenting strategies, such as yelling. Indeed, preliminary studies have suggested child evocative effects on CM (Choe et al., 2013; Combs-Ronto et al., 2009; Stith et al., 2009). Children exhibiting disruptive behaviors can be distressing and frustrating for their caregivers (McElroy & Rodriguez, 2008). If a caregiver is unable to cope with this stress (e.g., on top of IPV-related stress and lack of resources), the caregiver may be more likely to aggressively discipline the child (as an attempt to stop the behavior; Leve & Cicchetti, 2016; McElroy & Rodriguez, 2008; Trickett & Kuczynski, 1986). Therefore, it may be that high rates of CM in our study partially stem from the family dynamics in our sample of help-seeking mothers with children showing subclinical behavior problems. Comparisons of prevalence rates need to be interpreted within the frame of cultural background as well as the high vulnerability of our sample. However, our findings clearly call for greater recognition of high rates of emotional CM in these countries, potentially wrapped in normative perceived parenting strategies.

### Child Neglect

The rates of child neglect reported in our study (14%–21%) are comparable to estimates reported in a global meta-analysis (16%; Stoltenborgh et al., 2012) as well as the local ACE studies (7%–31%; Baban et al., 2013; Lesco et al., 2018; Raleva et al., 2013). These comparable rates may be partly explained by parental failure to meet the child´s need for food, which is an item consistently used to assess physical neglect across studies. Shortage of food supply and food insecurity affect many families in resource-poor countries. In fact, 31% of caregivers reported at least one form of hunger during the last 4 weeks, which was associated with a higher incidence of child neglect in our pilot study (Jansen et al., 2020). However, we must recognize that it is difficult to distinguish between poverty and deliberate withholding of food in studies of CM (Raleva et al., 2013).

### Risk Factors for Co-Occurrence of IPV and CM

We found that experience of CM during childhood of mothers and elevated levels of externalizing behavior problems of children were associated with increased likelihood of reporting co-occurrence of IPV and CM. Interestingly, mothers’ exposure to CM in their own childhood was associated with IPV in adulthood, as well as the co-occurrence of IPV and CM, but not with CM alone. This finding may support preliminary research, suggesting that IPV may be a potential mediating factor to explain the intergenerational transmission of CM. For example, recent studies found that the relationship between CM victimization in childhood and the potential for later CM perpetration in adulthood is mediated by maternal IPV exposure (Adams et al., 2019) as well as IPV-related PTSD symptoms (Anderson et al., 2018). Moreover, our findings support emerging evidence indicating that caregiver mental health problems, as well as those of children, play a key role in the development and maintenance of family violence. Bi-directional relationships between child behavior problems and CM or IPV are well established (e.g., Evans et al., 2008; Stith et al., 2009). Therefore, child behavior problems may constitute both a risk factor and an outcome of family violence. Although several studies have shown that caregiver and child mental health problems are associated with both IPV and CM (Guedes et al., 2016; Jouriles & Norwood, 1995; Margolin & Gordis, 2003), longitudinal studies are rare. Thus, prospective studies are warranted to shed light on how these factors contribute to potential spill-over effects across types of family violence.

With the exception of child behavior problems, we did not find any other contextual or external environmental stressors linked to the co-occurrence of IPV and CM. This is inconsistent with the theory that specific stressors and stressful circumstances may trigger aggressive behavior among family members, which may lead to different forms of family violence (e.g., Jouriles et al., 2008; Slep & O’Leary, 2001). Although preliminary studies from LMICs (Buffarini et al., 2021) show that contextual risk factors (e.g., low family income, neighborhood violence, and low maternal education) are associated with IPV and CM co-occurrence, most evidence have been derived from high-income settings. Thus, the apparent effects of stressors on either IPV or CM stated in the literature have not been sufficient to predict their co-occurrence in our sample from LMICs. These null findings must be critically appraised. Our caregiver sample was characterized by a range of contextual and environmental stressors per se, as defined within the scope of the study, including sampling families living in a LMIC who had children with elevated behavior problems and only those between the ages of 2 and 9 years. Results could in part be attributed to interacting sociocultural factors that we did not consider in our model but have been shown to be associated with family violence. For example, living in areas with high levels of male patriarchal control, discrimination, male dominance in the household, and societal acceptance of violence has especially received increasing attention in the research field of IPV and CM (Guedes et al., 2016). Our study—similar to most studies in the literature on CM and child behavior problems—leans on mother–child relationship data. To avoid “mother-blaming” tendencies in the literature, factors such as coercive control by male caregivers need to be recognized as well (Katz, 2015, p. 73). Coercive control comprises multiple behaviors involving the control and monitoring the “time, space and movement” of mothers and children (Katz, 2019, p. 48). It is often accompanied by intimidating behavior and the denial of financial or emotional resources (Katz, 2019; Lehmann et al., 2012; Stark, 2007). These behaviors are not just CM, but also lead to isolation of children from social support and extra-curricular activities, which in turn may contribute to the development of child behavior problems (Katz, 2015). Future studies, especially those that would allow cross-cultural comparisons in LMICs, should consider including more sociocultural risk factors (linked to emotional, social, and educational harm to mothers and children) in their models to reflect the complexity of family violence patterns.

The current study has several limitations. First, only mothers who reported elevated levels of child behavior problems were included in this study, so both samples were not representative of the overall population of mothers in North Macedonia, the Republic of Moldova, and Romania. In addition to being linked to IPV and CM, elevated levels of child behavior problems have been associated with a range of family adversities, including poverty and parental mental health problems (Deater-Deckard et al., 1998), which is likely to contribute to the strong associations and high rates of family violence that we found. Findings may be different for families with infants or adolescents as well. Second, our findings are based on cross-sectional data. No causal inferences on the associations found can be drawn. Third, to assess current CM, only one index child was considered during assessments. Future studies may benefit from employing child-reporting measures, considering data from both partners, and collecting longitudinal data to capture the extent and complexity of family violence. Furthermore, the newly translated measures of IPV and CM were tested in a feasibility study with only small sample sizes across countries (*n* = 50 in North Macedonia, *n* = 43 in the Republic of Moldova, and *n *= 47 in Romania; Jansen et al., 2021). More future studies with larger sample sizes are required for external validation. Our findings suggest that young children with elevated levels of behavioral problems constitute a vulnerable population for exposure to multiple forms of family violence. More research on evidence-based prevention programs is needed to develop and optimize programs specifically tailored to the needs of mothers in challenging family and regional contexts. Future longitudinal studies may consider more sociocultural factors, as well as the optimum periods for efforts that aim to break the cycle of family violence.

## Conclusion

In our sample of mothers with children showing increased behavioral problems, mothers exposed to IPV in the past month were more likely to maltreat their child in the same month. For practitioners, awareness and guidance on understanding the complexity of family violence and the intersections across types of violence are needed. Practitioners should be aware that, when detecting one form of family violence, there is a fair chance that there may be other forms of violence co-occurring in the same household that may also require attention. Our findings add to the literature on the transgenerational transmission of violence and indicate that maternal and child mental health problems play a crucial role in the cycle of family violence. This increases the knowledge base on IPV and various types of CM in Eastern Europe, especially in considering a particularly vulnerable sample of mothers seeking help for parenting. Our study provides critical evidence that targeted interventions are needed for disadvantaged mothers. Experience of CM during childhood by caregivers and externalizing behavior problems of their children are specifically associated with IPV and CM co-occurrence, indicating that these factors may need to be more strongly addressed in future preventive efforts. Thus, findings may guide future research and stakeholders in developing and implementing specially tailored programs that target more than one form of family violence.

## Supplemental Material

sj-docx-1-vaw-10.1177_10778012231188090 - Supplemental material for Co-Occurrence of Intimate Partner Violence Against Mothers and Maltreatment of Their Children With Behavioral Problems in Eastern Europe Click here for additional data file. Supplemental material, sj-docx-1-vaw-10.1177_10778012231188090 for Co-Occurrence of Intimate Partner Violence Against Mothers and Maltreatment of Their Children With Behavioral Problems in Eastern Europe by Antonia Brühl, Catherine L. Ward, Jamie M. Lachman, Heather M. Foran, Marija Raleva, Adriana Baban and Nina Heinrichs in Violence Against Women
