# Supplementary material for: Co-Occurrence of Intimate Partner Violence Against Mothers and Maltreatment of Their Children With Behavioral Problems in Eastern Europe
Source: Violence Against Women. 2023 Jul 20;29(12-13):2439–63. doi: 10.1177/10778012231188090 (PMC10496420; doi:10.1177/10778012231188090)
Supplement: sj-docx-1-vaw-10.1177_10778012231188090 - Supplemental material for Co-Occurrence of Intimate Partner Violence Against Mothers and Maltreatment of Their Children With Behavioral Problems in Eastern Europe [file sj-docx-1-vaw-10.1177_10778012231188090.docx]

**Supplementary Material**

**Supplementary Material A. Frequencies and prevalence rates for CM and IPV found in the ACE studies**

**Table 1**

*Frequencies and prevalence rates for CM and IPV in North Macedonia, Republic of Moldova, and Romania found in the ACE studies*

|  | North  Macedonia  *n (%)* | Republic of  Moldova  *n (%)* | Romania  *n (%)* |
| --- | --- | --- | --- |
| Child maltreatment |  |  |  |
| Physical abuse | 269 (21.1) | 167 (12.0) | 551 (26.9) |
| Emotional abuse | 139 (10.9) | 212 (15.01) | 488 (23.6) |
| Sexual abuse | 165 (12.9) | 59 (3.9) | 178 (9.0) |
| Physical neglect | 255 (20.0) | 109 (7.3) | 385 (18.5) |
| Emotional neglect | 391 (30.6) | 186 (12.9) | 548 (26.3) |
| Intimate partner violence | 129 (10.1) | 183 (13.1) | 359 (17.4) |

*Note.* IPV = Intimate partner violence in form of violence against the mother; North Macedonia (N = 664; Raleva et al., 2013); Republic of Moldova (N = 1534; Lesco et al., 2018); Romania (*N* = 2088; Baban et al., 2013).
